# Non-Cooperative Target Recognition by Means of Singular Value Decomposition Applied to Radar High Resolution Range Profiles ^†^

**DOI:** 10.3390/s150100422

**Published:** 2014-12-29

**Authors:** Patricia López-Rodríguez, David Escot-Bocanegra, Raúl Fernández-Recio, Ignacio Bravo

**Affiliations:** 1 Detectability and Electronic Warfare Laboratory, INTA, Torrejón de Ardoz, Madrid 28850, Spain; E-Mail: escotbd@inta.es; 2 Department of Applied Mathematics to the Telecommunication Technical Engineering, Polytechnic University of Madrid, Madrid 28031, Spain; E-Mail: rfrecio@euitt.upm.es; 3 Electronics Department, University of Alcala, Alcala de Henares, Madrid 28871, Spain; E-Mail: ibravo@depeca.uah.es

**Keywords:** NCTI, ATR, range profiles, SVD, synthetic database, actual measurements

## Abstract

Radar high resolution range profiles are widely used among the target recognition community for the detection and identification of flying targets. In this paper, singular value decomposition is applied to extract the relevant information and to model each aircraft as a subspace. The identification algorithm is based on angle between subspaces and takes place in a transformed domain. In order to have a wide database of radar signatures and evaluate the performance, simulated range profiles are used as the recognition database while the test samples comprise data of actual range profiles collected in a measurement campaign. Thanks to the modeling of aircraft as subspaces only the valuable information of each target is used in the recognition process. Thus, one of the main advantages of using singular value decomposition, is that it helps to overcome the notable dissimilarities found in the shape and signal-to-noise ratio between actual and simulated profiles due to their difference in nature. Despite these differences, the recognition rates obtained with the algorithm are quite promising.

## 1. Introduction

The goal of a non cooperative identification (NCI) system is to reliably identify unknown targets with no need to establish communication with them. These systems compare the observed target data with a database of potential targets in order to determine the closest match. High range resolution radar data have been shown to provide plenty of information to identify unknown targets [1–5]. A high resolution range profile (HRRP) is a one-dimensional radar image where the reflectivity of a target is projected onto the radar line of sight. Profiles are comprised of range bins and contain the distribution of the scattering centers of a target providing information about target structure such as its size, scatterers distribution and so on [6]; moreover, radar provides the ability to recognize targets at long distances and under any weather conditions, thus, the use of HRRPs for target identification has been a key research domain in the Defense industry and the radar automatic target recognition (ATR) community during the last two decades [2,3,7–11]. However, the use of HRRPs for classification purposes is still a challenging task due to the extreme within-class variability and the high dependency of the shape of these profiles with the target aspect angle.

Since the aircraft will be moving while collecting range profiles, the aspect angle and the distance between radar and aircraft will change. This leads to the appearance of three main sources of variation: speckle, rotational range migration (RRM) and translational range migration (TRM). Speckle occurs when the same range bin contains information about more than one scatterer. Then, a slight motion of the aircraft can cause the scatterer contribution to turn from constructive to destructive interference or vice versa causing the peak amplitudes to change rapidly between sequentially collected profiles. RRM is caused if HRRPs are collected during a wide rotation of the aircraft, making the scatterers move from one range bin to the other. These effects are referred to as target-aspect sensitivity. On the other hand, TRM is due to the translational motion of an aircraft which changes the distance between radar and target and causes scatterers to move from one range bin to the next. In this case, the relative distance between two scatterers does not change since they are all moved the same amount. This is known as the time-shift sensitivity, which causes a cyclically shift in consecutive profiles, implying that the position of the target signal within a HRRP sample vary with each measurement. Another effect that should be accounted for is the amplitude-scale sensitivity. It comes from the fact that the intensity of a HRRP is a function of the radar transmitting power, target distance, radar antenna gain, receiver gain, system losses and so on, thus, profiles measured by different radars or under different conditions will have different amplitude scales. Consequently, some preprocessing techniques are needed in order to deal with this target-aspect, time-shift and amplitude-scale sensitivity of a HRRP [2,3]. Despite these inconveniences, not only is much easier to obtain reliable range profiles than focused 2D radar images, such as inverse synthetic aperture radar (ISAR), but also, the computational time needed to obtain a recognition output is much shorter.

After the application of preprocessing techniques to improve the quality of profiles and in order to preserve relevant information, remove redundancies and capture significant attributes of aircraft in the range profiles, feature extraction techniques (including dimensionality reduction) are needed before the identification process takes place. With feature extraction, redundant information that range profiles may have is removed and only a reduced representation of data is needed to perform classification. Thus, non cooperative target recognition gets less computationally intensive and has potential for real time processing.

In the literature, different methods for feature extraction in HRRP have been studied. The traditional dimensionality reduction algorithms for HRRP recognition are based on a reconstruction model like principal component analysis (PCA) [3]. Wavelet transformation can also be applied as a feature selection method [12]. Hidden Markov models (HMMs) have been employed in [13] to statistically characterize the sequential information in HRRPs while the features are extracted via a RELAX algorithm. The differential power spectrum, which was originally used in speech recognition, is introduced to extract features from range profiles in [14]. Another common approach is feature extraction in the frequency domain and the use of the Fourier transformed range profiles as feature vectors [15,16]. Zyweck and Bogner [17] compute the dimensionality reduction with a linear discriminant function. As a time-shift invariant feature, bispectra have also been studied in [18,19], however the computational burden needed to compute the bispectra is too high. In [20], a dictionary learning algorithm based on K-SVD for sparse signal approximation is proposed as a new method for dimensionality reduction in radar target recognition.

Bhatnagar *et al.* [8] showed that using singular value decomposition (SVD), the eigenvectors corresponding to the *m* largest eigenvalues of the correlation matrix of range profiles constitute the optimal basis feature set in the minimum mean square error sense. Equivalent to PCA, SVD transforms a matrix into different subspaces, but instead of using the method as a reconstruction model, in the methodology presented here SVD is used in order to work in the transformed domain, *i.e.*, directly with eigenvectors and eigenvalues. Then, dimensionality is reduced to extract the main features of the range profiles and to reduce the unwanted information; additionally, this approach lightens the computational burden since there is no need to reconstruct the initial profile.

SVD has been also previously applied to image processing for image compression, image denoising or even for watermarking applications [21]; to reduce the noise in digital receivers [22]; to 3D object classification [23] where application of SVD is used in order to model static images as subspaces and reduce dimensionality, and also to target recognition with range profiles as in [8], where the application of SVD to ATR was first used (algorithm extended later in [24]); or in [25] where SVD is used for the reduction of noise. In this study, with the application of SVD, targets are modeled as subspaces so as to reduce dimensionality and to define metrics based on the angle between subspaces.

This paper is organized as follows: Section 2 first presents the construction of the database used for recognition followed by an introduction of the SVD technique and the definition of the proposed algorithms. Once the algorithms are introduced, the section gives an insight about the dataset used in the experiments. Section 3 provides a discussion and the results obtained with the proposed method and finally, Section 4 presents the conclusions and future work.

## 2. Methodology

### 2.1. Database construction

Traditionally, target recognition algorithms are validated via comparison of a test set of actual HRRPs with a database of potential targets previously collected via cooperative measurements [3,26]; on the contrary, there are some authors that validate their algorithms via comparison of a test set of predicted HRRPs with a database of profiles predicted also via electromagnetic simulations [8,27]. The principal disadvantage in proving the accuracy of the algorithms lies in the fact that comparison is done in the same domain, that is, actual measurements vs. actual measurements or simulated profiles vs. simulated profiles. In both cases, the test and training samples have similar nature and usually a similar high signal-to-noise ratio (SNR) resulting in good recognition results.

In a real hostile situation, e.g., at battle time, the range profiles of an unknown target are collected in a scene where high SNR cannot be guaranteed due to the measurement collection conditions like long radar distance, thus, actual target signatures will be less clear than those in the database; additionally, in order to guarantee the right recognition, the unknown target must have been previously measured in a similar aspect angle and configuration (pods, missiles, *etc*.) and loaded to the target database. Thus, comparison with actual measurements implies the previous collection of information from a great number of flying targets in different aspect angles and configurations and even so, the main problem lies in the fact that not all existing aircraft may have been measured. Therefore, the aircraft to be identified would unlikely be included in the database and so, it would be incorrectly classified. It is important to develop a recognition system with a database of targets that holds the generalization capability, that is, a database with information about the vast majority of existing targets in as many trajectories as possible. In order to obtain this wide database, its population with HRRP simulations is thought to be a good choice.

But, why is the use of predicted profiles as database interesting for classification? As noted, it is impossible to fill a database only with measured profiles since among other reasons, aircraft from hostile nations will never participate in measurement campaigns. Populating a database with synthetic target signatures has certain advantages: target signatures of any target in any aspect angle and configuration can be obtained with the use of radar cross section (RCS) prediction software so the database can be as wide as required, the database population is fast and low-cost and its update (addition of new targets, configurations or aspect angles) only implies CAD modeling and simulation instead of planning expensive and lengthy cooperative measurements campaigns. On the other hand it also has disadvantages: simulations are run in ideal environments, software simulation tools may not take into account all electromagnetic (EM) effects and aircraft models may not be exact replicas of real ones. These imply that synthetic signatures will be very clean compared to an actual measurement of the same target, hindering the identification process. Thus, the identification algorithms to be developed must be robust with the difference in shape and SNR between test and training samples.

According to that, in this paper identification of HRRPs coming from data of real in-flight targets is carried out by comparison with a database of simulated/synthetic HRRPs. This approach is barely applied in the open literature [2,28,29] but it is a very interesting field due to the ease in the database population and the fast evaluation of algorithms. The main drawback found, as noted, is that predictions have a very clean signature while actual HRRPs suffer from noise and other unwanted effects, making the recognition process similar to a real situation where collected profiles could be noisier than those in the database. In order to overcome the differences between HRRPs and to keep only the main features of a target, SVD [30] is applied to matrices of consecutive range profiles.

### 2.2. Singular Value Decomposition

SVD is a robust technique for the decomposition of any matrix into orthogonal basis spaces [30]. With SVD it is possible to find the best approximation of the original data points using fewer dimensions. Let *X* ∈ ℜ*^N^*^×^*^M^* be a matrix of consecutively collected real HRRPs of dimension *N* × *M* (assuming *N* > *M*), with *M* being the total number of profiles and *N* the number of range bins. There exist orthogonal matrices

(1) $$U=[u_{1},\ldots ,u_{N}]\in {ℜ}^{N\times N}$$

(2) $$V=[v_{1},\ldots ,v_{M}]\in {ℜ}^{M\times M}$$

such that

(3) $$U^{T}XV=\text{diag}(\sigma_{1},\ldots ,\sigma_{p})\in {ℜ}^{N\times M};p=\min\{N,M\}$$

where *σ*_1_ ≥ *σ*_2_ ≥ … ≥ *σ_p_* ≥ 0 are the singular values of *X*, *diag* stands for diagonal matrix and vectors *u_i_* and *v_i_* are the *i*th left and *i*th right singular vectors of *X* respectively. The left singular vectors in *U* span the orthogonal basis space in the range domain while the right singular vectors in *V* span the basis space in the angle domain. Larger singular values, *σ_i_*, imply larger contribution of the corresponding singular vector in forming the target signal. The Eckhart and Young theorem [30] guarantees that the top singular vectors with the highest singular values provide the best approximation of the data. Thus, the *N*-dimensional vector space (when referring to matrix *U*, or *M*-dimensional, referring to *V*) can be divided into two subspaces, a dominant subspace, namely the *signal subspace*, and a subdominant subspace, namely the *noise subspace*. Therefore, the singular vectors associated with the largest singular values are the basis that span the *signal subspace* while the rest are the basis that span the *noise subspace* and will be discarded in the identification process.

### 2.3. Algorithm Definitions

Since HRRPs present the target reflectivity into the range domain, only the left singular vectors will be used in the identification process. Taking into account the singular values, *σ_i_* and setting an energy threshold *η* (0 < *η* < 1) as in Equation (4), the *signal subspace* is defined as the *K* most significant *u_i_* singular vectors, while the *noise subspace* is discarded.

(4) $$\frac{\sum_{i=1}^{K}\sigma_{i}}{\sum_{i=1}^{p}\sigma_{i}}\geq \eta$$

**Figure 1.**
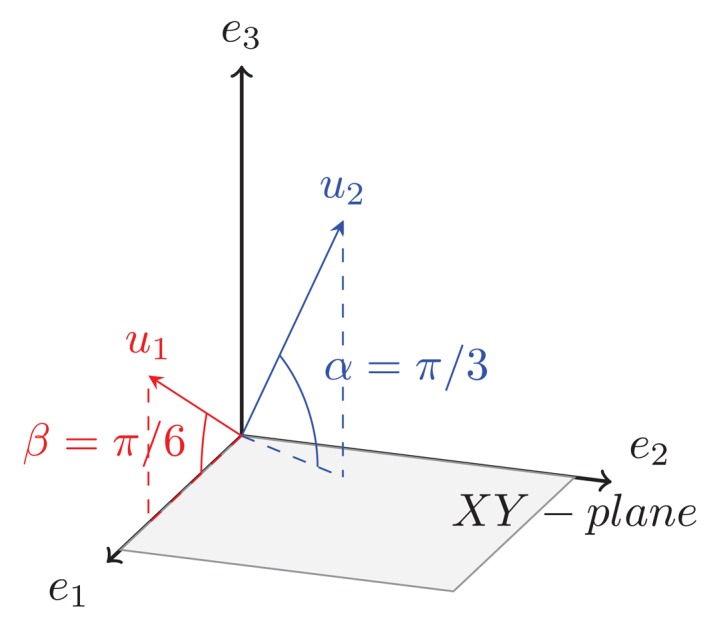
The *signal subspace* is defined by vectors *e*_1_ and *e*_2_.

In order to clarify the metrics used in this research, let us introduce the simplified concept of subspace division and angle between subspaces shown in the example of Figure 1. In this paper we call test set to the actual profiles to be identified and training set to the data that populate the synthetic database of already known targets.

Let vectors *e*_1_, *e*_2_ and *e*_3_ in Figure 1 be the left singular vectors, as defined in Equation (1), obtained after applying SVD to the test set to be identified. Imagine that the threshold *η* is set to 0.95. Assuming that according to their associated singular value the 95% of the energy is focused on *e*_1_ and *e*_2_, then these are defined as the *K* = 2 first left singular vectors that form the basis of the *signal subspace*, while *e*_3_ is discarded; hence, the *signal subspace* of the test set in this example corresponds to the XY plane. After the application of SVD to the training set, vectors *u*_1_ and *u*_2_ in Figure 1 are obtained as its left singular vectors as defined in Equation (1). In order to know the level of dependency of these vectors to the test set *signal subspace*, the angle between them and their projection onto it is obtained. Notice that *u*_1_ is closer to the XY plane than *u*_2_, in this example the angle found for *u*_1_ is *β* = *π*/6 while the angle found for *u*_2_ is *α* = *π*/3; the smaller the angle, the closer to the subspace.

Denoting *X^R^* as the *signal subspace* of the test set containing its *K* first left singular vectors and $u_{i}^{s}$ as the *i*th left singular vector of the training set corresponding to target *s*; function *F*1*_s_*, given by Equation (5), is defined as the accumulated angle between a singular vector $u_{i}^{s}$ in the training set and the *signal subspace* of the test set, where $∠(X^{R},u_{i}^{s})$ is the angle between the test set *signal subspace* and each singular vector of the training set as stated in [30].

(5) $$F1_{s}(k)=\sum_{i=1}^{k}∠(X^{R},u_{i}^{s});k=1,\ldots ,K$$

*F*1*_s_* shows the evolution of the angle formed by each synthetic singular vector and its projection onto the *signal subspace* resulting in a monotonically increasing function. The recognized aircraft, *s*, will be the one with the lowest final value of *F*1*_s_*. In the case of function *F*1*_s_*, the angle of every singular vector in the training set contributes equally to the final result, *i.e.*, singular vectors are equally important. Imagine our training set consists of *S* = 2 different aircraft, *A* and *B*, and let vectors *u*_1_ and *u*_2_ in Figure 1 be the left singular vectors obtained for these aircraft after applying SVD to their respective matrices of range profiles, such that:

(6) $$\begin{array}{l} \text{aircraftA}=\{\begin{array}{l} u_{1}^{A}=u_{1}\Rightarrow \sigma_{1}^{A}=0.8\\ u_{2}^{A}=u_{2}\Rightarrow \sigma_{2}^{A}=0.2 \end{array}\\ \text{aircraftB}=\{\begin{array}{l} u_{1}^{B}=u_{2}\Rightarrow \sigma_{1}^{B}=0.8\\ u_{2}^{B}=u_{1}\Rightarrow \sigma_{2}^{B}=0.2 \end{array} \end{array}$$

where the superscript represents the aircraft *s* (*s* = *A*, *B*; *S* = 2) to which the singular vectors and singular values are related. With the application of function *F*1*_s_* there would be confusion in the identification result since:

(7) $$F1_{A}(K)=F1_{B}(K)=\pi /3+\pi /6$$

However, their associated singular values reveal that not all singular vectors in the training set have the same importance since the energy is focused on the top ones. This means that the obtained angle between $u_{i}^{s}$ and *X^R^* should be weighted in a way that the singular value $\sigma_{i}^{s}$ associated with $u_{i}^{s}$ sets the importance of this angle in the final solution. For instance, angles of *π*/2 mean that the singular vector is orthogonal to the subspace. Thus, obtaining results of this order when $u_{i}^{s}$ is associated with a high singular value would mean that the aircraft (*s*) to be recognized will not belong to that class. On the contrary, if an angle of *π*/2 is obtained with a vector with a very low singular value it will not contribute to a great extent to the final solution. According to this, function *F*1*_s_* is modified in order to add some kind of weighting to the angles between subspaces found. Function *F*2*_s_* given in Equation (6) returns the accumulated weighted angle *F*2*_s_* between the *signal subspace* of the test matrix and the singular vectors *u_i_* for each synthetic aircraft *s* in the training set.

(8) $$F2_{s}(k)=\frac{1}{\sum_{j=1}^{K}\sigma_{j}^{s}}\sum_{i=1}^{k}\sigma_{i}^{s}\cdot ∠(X^{R},u_{i}^{s});k=1,\ldots ,K$$

In Equation (6) $\sigma_{i,j}^{s}$ are the *K* first singular values associated to each synthetic aircraft in the training set and, as in Equation (5), $∠(X^{R},u_{i}^{s})$ is the angle between the test set *signal subspace* and each singular vector of the training set. Finally, the algorithm decides the test sample belongs to the target that minimizes the cost function (6). In the previous example, in contrast to *F*1*_s_* the application of Equation (6) would result in the identification of aircraft A since:

(9) $$\begin{array}{l} F2_{A}(K)=1\cdot (0.8\cdot \frac{\pi }{6}+0.2\cdot \frac{\pi }{3})\\ F2_{B}(K)=1\cdot (0.8\cdot \frac{\pi }{3}+0.2\cdot \frac{\pi }{6}) \end{array}\}F2_{B}(K)>F2_{A}(K)$$

So, by applying SVD to HRRP matrices and selecting the singular vectors of the *signal subspace* not only is the reduction of the amount of data achieved, but the identification process is also improved due to the rejection of the *noise subspace* and the use of the transformed domain.

### 2.4. Datasets

For the purpose of classification, two collections of range profiles are used in this research, a test set and a training set. The test set consists of measured HRRPs from a civil aircraft measurement campaign [31], while the training set is made of a collection of simulated HRRPs using the RCS-prediction code FASCRO [32]. FASCRO predicts the monostatic RCS of a target based on high frequency (HF) techniques (Physical Optics, PO, and Physical Theory of Diffraction, PTD).

**Figure 2.**
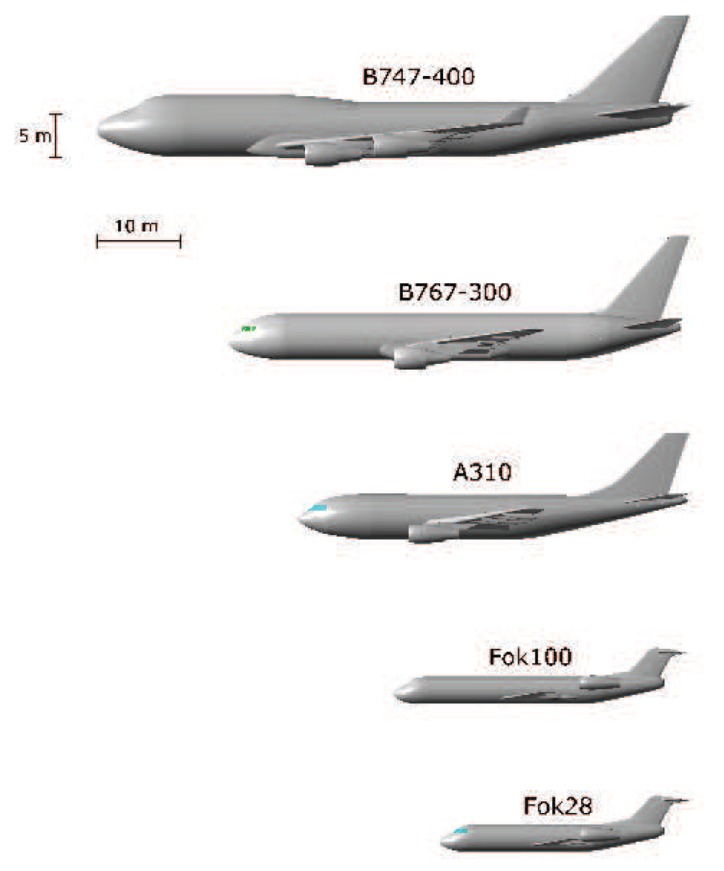
CAD models used for the RCS predictions.

The test set includes measurements of 5 civil in-flight aircraft in different flighpaths, the Boeing 747-400, the Airbus A310, the Boeing B767-300, the Fokker 28 and the Fokker 100, a large, two medium and two small-sized aircraft. These data were collected with the FELSTAR S-Band radar at TNO-FEL located in The Netherlands. During acquisition, information from a secondary radar was available providing the target type and an estimate of its flightplan. The error on the estimated aspect angle of the aircraft does not exceed 5 degrees in both azimuth and elevation. Rapid changes in elevation, or if the aircraft was making a long turn in its trajectory, would affect the profiles, nevertheless, in the measurement campaign, only targets under conditions of no long turns nor approaching or leaving an airport runway were measured. Moreover, the measured profiles are free of influences of radial velocity since the FELSTAR radar used a velocity tolerant waveform (the times at which the pulses are transmitted are chosen such that the resulting range profiles are focused irrespective of the velocity). Since the generalization capability is sought, for the creation of the training set the CAD models of the same 5 aircraft have been developed at INTA (Spanish National Institute for Aerospace Technology) and the profiles have been predicted by FASCRO using the information of the estimated aspect angles given in the test set. Figure 2 shows the CAD models used to obtain the simulated profiles.

**Figure 3.**
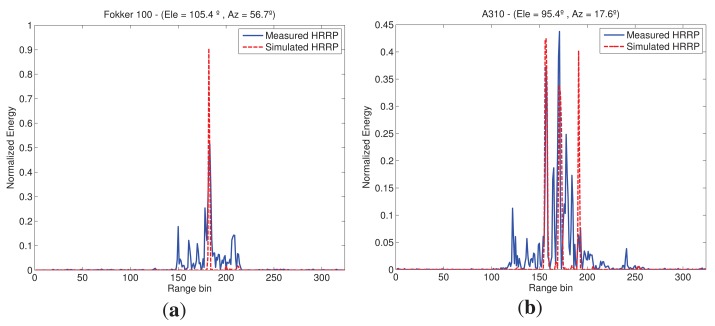
Difference between actual and synthetic range profiles. (**a**) Measured *vs*. Simulated Profile—F100; (**b**) Measured *vs*. Simulated Profile—A310.

The training set has been developed considering every aircraft as perfect electric conductors (PEC) with no protruding elements. It is also worth noting that FASCRO, as it uses high frequency techniques, does not take into account all EM effects when predicting RCS. Therefore, noticeable difference between test and training sets is expected hindering the identification process. An example of the differences between synthetic and actual profiles can be found in Figure 3 where profiles at a certain aspect angle of two types of aircraft are depicted.

One source of error in the resemblance is the target aspect estimate. As mentioned before, the synthetic profiles are predicted in the same aspect angles than the measured ones, however, there exist an error in both azimuth and elevation on the predicted aircraft orientation and, as said, the aspect angle under which the aircraft is seen affects the shape of the HRRP Another observation is that the amplitudes of the profiles in Figure 3 do not match very well; CAD modeling defined as PEC is only a first approximation to the actual scattering mechanisms and is therefore, likely to produce inaccuracies in the HRRP predictions. Moreover, although in both cases amplitude normalization is applied, the SNR difference between them is noteworthy; as seen in Figure 3, actual profiles are noisier between peaks than the simulated ones, which influences the amplitudes when normalizing; additionally, as no noise-power is present in the synthetic profiles its normalization pushes the signal components to higher values. Finally, another reason of the HRRP differences is that several scattering processes that occur in reality are not accounted for in FASCRO since it is based on HF techniques. A full wave EM software would be needed to properly run all these effects with the associated increase in time and memory requirements.

A total number of 21 trajectories are considered in this study for classification. With the purpose of avoiding RRM, each trajectory has been split into frames, each frame (sequence of collected profiles ordered in time and time-shift compensated [33] in order to palliate TRM) cover approximately a variation of 2.5° in azimuth in the aircraft aspect angle. If two profiles have aspect angles whose difference is less than Δ*α_RRM_* = Δ*R*/*T_D_*[*rad*] the profiles do not differ due to RRM [34]. According to the FELSTAR specifications and the longest aircraft in the database, in this study Δ*α_RRM_* = 0.3°. With the division of trajectories into frames, the effects of RRM are compensated since it is guaranteed that no pair of consecutive profiles exceed this Δ*α_RRM_*.

**Figure 4.**
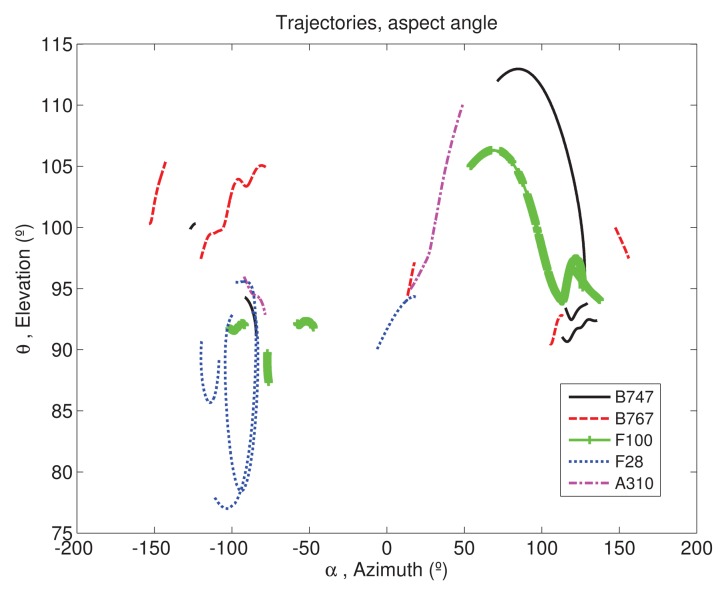
Aspect angles of the measured HRRP.

Figure 4 shows the aspect angles of the trajectories used in the process, where nose-on aspect angles corresponds approximately to (*θ* = 90°, *α* = 0°). Note that since the method pretends to be validated for any aspect angle, this study is not only focused on trajectories in nose-on, any trajectory is valid.

**Figure 5.**
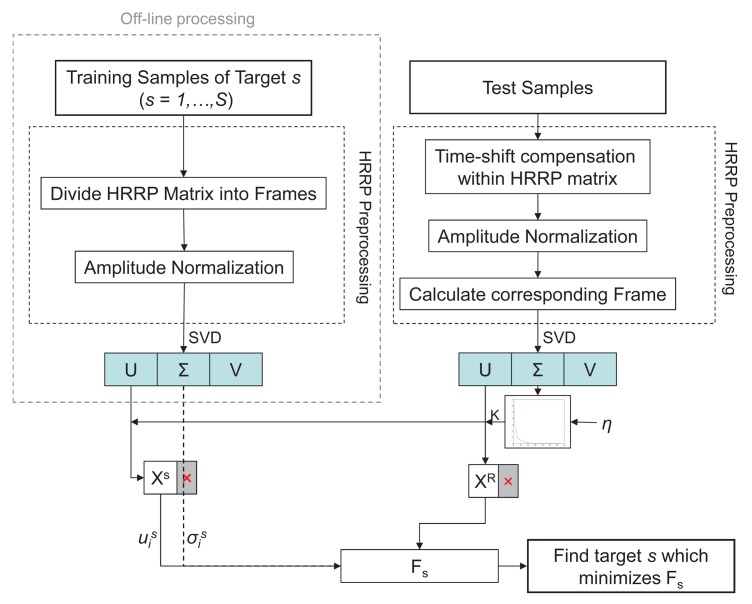
Flow chart of the proposed recognition algorithm.

Most studies tend to classify one profile at a time [8,10,24]. Here, the identification is carried out using a sequence of profiles so as to have more information about the position of the scattering centers of the target and their evolution along its trajectory. As stated, frames of synthetic profiles are the same as the actual profiles meaning that they are predicted in the same estimated aspect angles as the profiles in the test set. Figure 5 shows the flow chart of the recognition algorithm proposed in this paper, where *S* is the number of different targets in the database, (*S* = 5), *F_s_* can be any of the two metrics (*F*1*_s_* or *F*2*_s_*) and the synthetic singular values ( $\sigma_{i}^{s}$) are only needed when the chosen metrics is *F*2*_s_*.

When a test sample is to be identified, an HRRP preprocessing stage should be carried out before feature extraction in order to mitigate the target-aspect, time-shift and amplitude-scale sensitivity of the profiles. Time-shift compensation is carried out with an alignment of profiles. The amplitude-scale sensitivity is compensated with a normalization to unit energy of each HRRP by applying L2-norm. In order to offset the target-aspect sensitivity the trajectories are divided into small frames with a variation of at most 2.5° in azimuth. So, in the preprocessing stage of the test set, one must find the corresponding frame into which the test sample is included. The average profile of a real HRRP frame can also reduce the target-aspect sensitivity of real HRRPs. In fact, the average vector of a frame and the first principal component of the correlation matrix of the same frame are similar [3]. Accordingly, instead of calculating the mean vector of a frame, the further application of SVD will help reduce the target-aspect sensitivity.

In the case of the training samples, their preprocessing stage differs from the former in two substages. First, the frames in which trajectories are divided into are defined in this stage, and second, time-shift compensation is not needed since the profiles come from ideal simulations where the aircraft motion is already compensated. After the preprocessing stage, there still exist some sources of variation between profiles of the same and different aircraft, mainly caused by noise, approximation of CAD models as well as software prediction errors. By applying SVD at this point, it is possible to separate the essential information from the redundant one.

As noted, the preprocessing and SVD of the training samples is done off-line, which means a reduction in the computational burden since the singular values and vectors of the potential targets will be already loaded in the database. Taking into account the singular values of the test samples and setting an energy threshold *η* as in Equation (4), the *signal subspaces* of both sets are defined as the *K* most significant *u_i_* singular vectors (*X^S^* and *X^R^* in the figure), and the *noise subspaces* are discarded resulting in a dimensionality reduction. Finally, using the defined *signal subspaces* the algorithm will identify the test sample as the target *s* that minimizes the chosen metrics (*F*1*_s_* or *F*2*_s_*).

## 3. Experimental Results

As presented in Section 2.4 the 21 trajectories are split into frames. For each frame and trajectory, the test and training sets are defined as matrices of range profiles of size *N* × *M*, with *N* = 324 number of range bins and *M* number of profiles. The number of profiles on each set depends on the trajectory and frame chosen; as Figure 4 shows, some trajectories have more rapid variation in azimuth than others and thus, to capture a variation of 2.5° in azimuth a less number of profiles will be needed. It also affects the number of *K* eigenvectors taken as *signal subspace*, as the number of profiles in a set decreases, the energy of the eigenvectors obtained by SVD is concentrated in a less number of eigenvectors. After the split of the trajectories, a total number of 175 frames are obtained for classification; 42 corresponding to aircraft class B747, 43 to F100, 32 to B767, 38 to F028 and 20 to A310. Recognition rates applying function *F*1*_s_* for the classification of the 175 frames of the test set with different energy thresholds can be found in Table 1.

The results given in Table 1 are obtained by simply computing the accumulated angle between subspaces as in Equation (5) and they show a very high error rate when comparing actual *vs.* synthetic profiles, that is, when comparing HRRP with different shapes and nature. As it can be seen, the lower the percentage of energy taken as *signal subspace* the better the average recognition rate, it rises from 56% when *η* = 0.99 to 65.1% when *η* = 0.85. If a high threshold is chosen (*η* = 0.99) almost all the eigenvectors are considered as *signal subspace*, that is, the *signal subspace* can contain information that actually belongs to the *noise subspace*. That is the reason why a reduction in the threshold returns better recognition rates, because the defined *signal subspaces* will be more accurate and the noise information will be truly discarded.

Nonetheless, there is a limit when choosing *η*; if it is too low the *signal subspace* will be composed of very few singular vectors and the decision interval is very small, that is, the metrics' final results for each aircraft are very close, which makes it difficult to distinguish between the winning aircraft and the following one even when the identification is correctly accomplished. So, when choosing *η* a parametric sweep should be executed in advance and a trade-off between recognition rate and decision interval should be taken into account. Consequently, in this study the chosen energy threshold is set to 85% (*η* = 0.85). According to Table 1, the recognition rates found for *F*1*_s_* are not high enough to be considered as a good classification; thus, in order to improve these results the need for using the singular values as weights in the cost function, as in *F*2*_s_*, is evident.

In Table 2 the recognition rates of the 175 frames in the test set obtained with *F*2*_s_* with different energy thresholds are presented. Also in this case, the recognition rates are enhanced with the decrease of the percentage of energy taken as *signal subspace*; in this experiment, the average recognition rate obtained when *η* = 0.99 is 75.4% and it rises to 82.3% when *η* = 0.85, but as noted, a lower limit in *η* must be set. As expected, the recognition rates have been improved with the addition of weighting elements in the metrics' definition.

**Figure 6.**
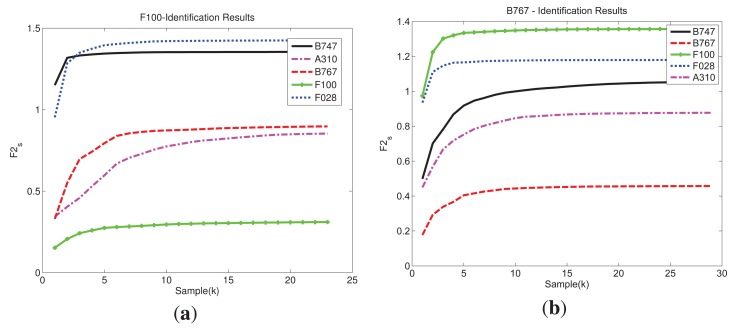
Example of identification results for two different aircraft in two different trajectories with a threshold of *η* = 0.85. (**a**) F100—identification results; (**b**) B767—identification results.

Figure 6 depicts an example of the identification curves obtained with function *F*2*_s_* for two different measurements in the test set. Each one is compared against the 5 synthetic aircraft in the database for the respective frame with a threshold of *η* = 0.85. As seen, the curves have a monotonically increasing tendency until they eventually reach a point of saturation; from that point on, the synthetic singular vector $u_{i}^{s}$, due to its corresponding singular value, does not add almost any new information to the recognition process. Figure 6a shows the recognition results of a F100 in a frame. The chosen threshold results, for that specific frame, in *K* = 23 singular vectors out of 83 that hold the 85% of the total energy and define the *signal subspace*. In this case, as F028 and F100 have similar geometry one could expect *F*2*_s_* to result in a similar value. However, in this example, the final values of these two aircraft differ the most. The geometric configuration of an aircraft has to do with the information returned from it but there are other factors that influence the formation of a HRRP. For a specific aspect angle, each range bin has information about all the scattering effects. These effects, as said in previous sections, are sometimes constructive, sometimes destructive, and make HRRPs very variable. This can cause, in a specific aspect angle, aircraft with similar configuration to be quite different. Additionally, as noted, FASCRO does not take into account all EM effects and this may have a more notable effect in some angles than in others. In order to illustrate that not always similar aircraft return very different results, Figure 6b, shows the recognition results of a B767 in a specific frame. Here, the *K* = 29 first singular vectors out of 132 hold the 85% of the energy and define the *signal subspace*. The aircraft that minimizes *F*2*_s_*(*K*) is the recognized target, therefore, in both examples of Figure 6 the identification is correctly accomplished.

Comparing the results of function *F*2*_s_* (Table 2) with the results of *F*1*_s_* (Table 1), each aircraft has obtained better recognition results when the singular values are used as weights in the cost function; this improvement in the recognition rates is more evident with a higher energy threshold, e.g., when *η* = 0.99 classification with *F*1*_s_* returns an average recognition rate of 56% while classification with *F*2*_s_* obtains a 75.4%, almost a difference in 20 percentage points.

When the threshold is set to be *η* = 0.85 this experiment shows a total average recognition rate of 82.3% with *F*2*_s_*, that is to say a 17.7% of error rate. This implies not only an improvement in the identification rate for a particular aircraft, but also an improvement in the global recognition rate of more than 15 percentage points (from 65.1% with *F*1*_s_* to 82.3% with *F*2*_s_*), enhancing the global recognition performance of the system when weighting elements are used. It is worth noting, one more time, the lack of resemblance between measured profiles and synthetic ones. As noted, actual profiles suffer from noise and unwanted information while synthetic ones, since they are run in an ideal environment without considering all electromagnetic effects and with CAD models which are not an exact replica of real aircraft, have a very high SNR and the signature is much clearer.

Real aspect angles are, at the most, 5° different from the estimated ones. This discrepancy is another add-on to the profiles dissimilarity and proves that, since recognition rates are up to 80%, SVD extracts the main information of a target along a trajectory near to the one that an aircraft is really following, *i.e.*, in order to obtain a fairly good recognition rate it is enough with the comparison of profiles in a surrounding of the real trajectory. If the estimated aspect angles were very accurate to the real ones in the trajectory an increase in the results would be expected.

Therefore, can it be affirmed that 82.3% is a good average recognition rate when identifying actual profiles by means of a synthetic database? Studies like the one presented in [3], which presents a method based on PCA for the recognition of complex measured HRRPs, obtain a high recognition rate of around 91%. However, they identify measurements from 3 flying airplanes with a database of actual measurements. That is, the identification is carried out between data of the same nature and with a small set of measurements, while the study presented in this paper identify data of different nature and with a quite bigger database. Yet, the method in [3] only outperforms *F*2*_s_* in 9 percentage points. On the other hand, among the studies in the open literature where identification of HRRPs of different nature is studied, the one in [2], although focused on translational motion compensation methods, carries out a preliminar study in recognition of predicted profiles previous to the classification of actual measurements with a synthetic database. Results show that when comparing simulated with simulated profiles the overall recognition rate reached 98%, however, when identifying actual aircraft measurements with predictions, the average recognition rate decreased to 70%. In both experiments the database is made of a collection of simulated range profiles of 5 aircraft models. As expected, the identification of predictions using predictions as a database clearly outperforms the identification rates shown in Table 2. However, in a similar scenario, where the experiment in which comparison has taken place between actual and simulated profiles, results in [2] are outperformed by 12 percentage points with the algorithm presented in this paper (82.3% *vs.* 70%).

The intention of this comparison is to check whether the recognition rates obtained here are close to those presented so far in the literature. Thus, it can be concluded that despite the obstacles found, recognition is accomplished with a good rate.

## 4. Conclusions

In this paper, a methodology for HRRP target recognition based on Singular Value Decomposition is shown. As noted, the main drawback of using actual measurements against simulated ones is the lack of similarity between range profiles, making identification not an easy task. Due to the extraction of the main information by reducing dimensionality, SVD not only helps to overcome these difficulties but also to reduce the computational burden since it is not necessary to store the unwanted information. Two methods based on SVD have been presented and compared. It has been proved that finding the angle between singular vectors and signal subspaces is not sufficient for obtaining good recognition performance. Nevertheless, the addition of a weighting element (singular values) in the cost function produces a rise in the identification rates, implying that recognition performance has been improved by introducing weighting. Considering the differences in nature of the test and training sets used in this research, the identification results obtained with the weighting method are quite promising and future experiments with larger sets are expected to be conducted in order to prove the accuracy of the method proposed here.

## Figures and Tables

**Table 1.** Average recognition rates with *F*1*_s_*.

**Class**	***η* = 0.99**	***η* = 0.9**	***η* = 0.85**
**B747**	54.8 %	57.1%	61.9%
**B767**	43.8%	87.5%	75.0%
**A310**	40.0%	55.0%	55.0%
**F100**	62.8%	46.5%	55.8%
**F028**	68.4%	73.7%	76.3%

**AVER. RECOGNITION RATE**	56.0%	63.4%	65.1%

**Table 2.** Average recognition rates with *F*2*_s_*.

**Class**	***η* = 0.99**	***η* = 0.9**	***η* = 0.85**
**B747**	90.5%	90.5%	92.9%
**B767**	59.4%	75.0%	78.1%
**A310**	85.0%	85.0%	90.0%
**F100**	65.1%	72.1%	72.1%
**F028**	78.9%	81.6%	81.6%

**AVER. RECOGNITION RATE**	75.4%	80.6%	82.3%
